# Current state-of-the-art on ganglioside-mediated immune modulation in the tumor microenvironment

**DOI:** 10.1007/s10555-023-10108-z

**Published:** 2023-06-02

**Authors:** Irene van der Haar Àvila, Britt Windhouwer, Sandra J. van Vliet

**Affiliations:** 1 Department of Molecular Cell Biology and Immunology, Amsterdam UMC location Vrije Universiteit Amsterdam, De Boelelaan, 1117 Amsterdam, the Netherlands; 2 Cancer Biology and Immunology, Cancer Center Amsterdam, Amsterdam, the Netherlands; 3 Cancer Immunology, Amsterdam Institute for Infection and Immunity, Amsterdam, the Netherlands

**Keywords:** Gangliosides, Shedding, Tumor immunity, Glycosylation

## Abstract

Gangliosides are sialylated glycolipids, mainly present at the cell surface membrane, involved in a variety of cellular signaling events. During malignant transformation, the composition of these glycosphingolipids is altered, leading to structural and functional changes, which are often negatively correlated to patient survival. Cancer cells have the ability to shed gangliosides into the tumor microenvironment, where they have a strong impact on anti-tumor immunity and promote tumor progression. Since most ganglioside species show prominent immunosuppressive activities, they might be considered checkpoint molecules released to counteract ongoing immunosurveillance. In this review, we highlight the current state-of-the-art on the ganglioside-mediated immunomodulation, specified for the different immune cells and individual gangliosides. In addition, we address the dual role that certain gangliosides play in the tumor microenvironment. Even though some ganglioside species have been more extensively studied than others, they are proven to contribute to the defense mechanisms of the tumor and should be regarded as promising therapeutic targets for inclusion in future immunotherapy regimens.

## Introduction

Glycosylation is the most abundant covalent modification that proteins and lipids undergo in living organisms [1]. Glycosylation occurs post-translationally in case of proteins, and post-synthesis for lipids and is highly diverse, in terms of glycan structures, and also varies with cell type, cellular activation, and during disease. Also, malignant transformation leads to an aberrant glycosylation profile [2]. One of the most frequently observed glycosylation changes in cancer is an increased expression of sialic acids, which negatively correlates to disease outcome and patient survival [3]. Sialic acids are terminal nine-carbon sugar residues present on mammalian glycoproteins and glycolipids. Sialylated glycosphingolipids, or gangliosides, are expressed throughout the entire human body, but are most abundant in the brain and nervous system. Due to their location in the outer leaflet of the plasma membrane, gangliosides participate in cell-cell and cell-matrix interactions and are able to modulate signal transduction of receptor tyrosine kinases (RTKs) through their association with lipid rafts. For example, tyrosine phosphorylation of epidermal growth factor receptor is inhibited by a variety of gangliosides [4]. In addition, certain pathogens, including malaria parasite *Plasmodium falciparum*, employ gangliosides to gain entry into the host cell and initiate infection [5, 6]. Gangliosides are likewise crucial in the protection of host structures against the autologous immune system by protecting host cells and tissues from complement attack and autoimmune responses [5, 7]. Even under healthy conditions, gangliosides are released and taken up by neighboring cells, possibly to coordinate signaling responses across cells and tissues [8].

Altered ganglioside expression has been linked to several pathological processes and is known to promote tumor initiation and progression [9]. Gangliosides are abundantly present in the tumor microenvironment (TME), as they are secreted by tumor cells in the form of micelles, monomers, and membrane vesicles [10]. Their glycan profiles are frequently disturbed during cancer progression and can therefore be used as tumor biomarkers [11]. Already in the 1980s, the shedding of tumor gangliosides was evident, showing a clear immunosuppressive activity *in vitro* and *in vivo* [12]. Tumor gangliosides are considered to have tumor-promoting properties and to stimulate tumor progression *in vivo* [13] by promoting cell motility, angiogenesis, and metastasis [11, 14]. Moreover, gangliosides are key modulators of signaling through tyrosine kinases and suppressors of immune surveillance against the tumor [15]. Therefore, the shedding of gangliosides by tumor cells has been strongly correlated to disease progression and lower survival rates [16]. Nevertheless, some gangliosides show anti-tumorigenic properties and differences in ganglioside function appear to vary between different tumor types [17]. In this review, we summarize the current state-of-the-art on ganglioside-mediated immune modulation in the tumor microenvironment, focusing on the expression and shedding of gangliosides by tumors, as well as their interactions with different immune cell subsets.

## Healthy synthesis of gangliosides

**Fig. 1:**
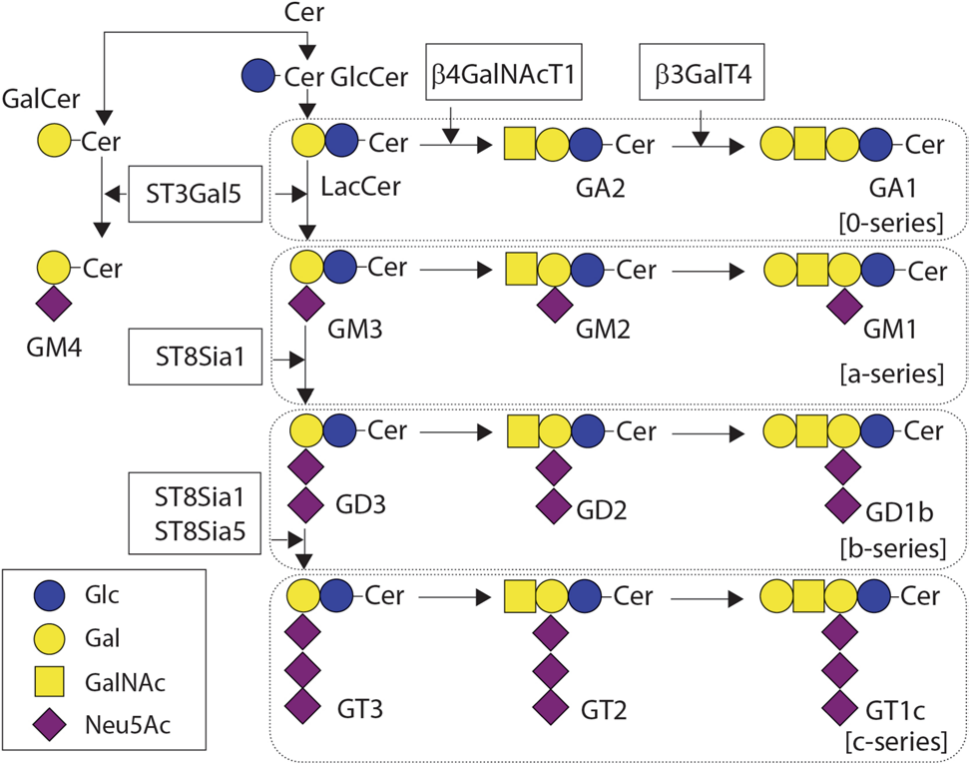
Schematic overview of the ganglioside structure and biosynthesis. Gangliosides are generated from the precursor LacCer or GalCer after addition of sialic acids by different sialyltransferases. Cer, ceramide; Lac, lactosyl; Gal, galactose; Glc, glucose; GalNAc, N-acetylgalactosamine; Neu5Ac, N-acetylneuraminic acid (sialic acid); ST3Gal5, ST3 βgalactoside α-2,3-sialyltransferase 5 (GM3 synthase); ST8Sia1, ST8 α-N-acetyl-neuraminide α-2,8-sialyltransferase 1 (GD3 synthase); ST8Sia5, ST8 α-N-acetyl-neuraminide α-2,8-sialyltransferase 5 (GT3 synthase); β4GalNAcT1, β-1,4-N-acetylgalactosaminyltransferase 1 (GM2/GD2 synthase); β3GalT4, β-1,3-galactosyltransferase 4 (GM1/GA1/GD1 synthase). Adapted from Inokuchi et al. [19]

Glycosphingolipids are formed through the stepwise addition of sugars by glycosyltransferases, first to ceramide and subsequently to the growing glycan structures, thereby generating a wide range of glycolipids. Gangliosides are a specific class of glycosphingolipids characterized by the addition of one or more sialic acids. Their synthesis starts in the endoplasmic reticulum and the further elongation occurs in the Golgi [18]. Gangliosides are classified into groups according to the number of sialic acid residues attached (*mono*, *di*, *tri*) and the order of migration of the gangliosides on thin layer chromatograms (GM3 > GM2 > GM1). The addition of one sialic acid to the precursor of most gangliosides, lactosylceramide (LacCer), generates GM3 and after addition of other sialic acids also GD3 and GT3. Each of these gangliosides then becomes an acceptor for another *N*-acetylgalactosamine (GalNAc) residue, generating the GM2, GD2, and GT2 lipids. Once the GalNAc is added, further addition or removal of sialic acid residues no longer occurs, thus committing the gangliosides to the “a-series” (one sialic acid), “b-series” (two sialic acids), or “c-series” (three sialic acids) (Fig. 1) [14]. After completion, the gangliosides are transferred to the outer leaflet of the plasma membrane, via vesicular delivery, where they are positioned facing the extracellular environment. Gangliosides are present in all cells; however, the exact ganglioside expression patterns differ per cell type and are dependent on the expression and intracellular distribution of the specific glycosyltransferases required for their biosynthesis [20].

## Shedding and expression of gangliosides in cancer

An altered ganglioside metabolism contributes to pathological conditions, such as cancer, autoimmune disease, and inflammatory disorders, especially if these diseases originate in the neurological system. For example, mutations in GM3 synthase have been identified as the cause of an autosomal recessive infantile-onset epilepsy syndrome [21]. The formation of anti-ganglioside antibodies has been related to Alzheimer’s disease, Parkinson’s disease, and Guillain-Barré syndrome [22–24].

During malignant transformation, the ganglioside repertoire is distinctly changed, due to alterations in the expression levels of glycosyltransferases involved in ganglioside synthesis [11]. These alterations vary from overexpression of certain gangliosides, loss of expression for others, truncated structures, accumulation of precursors, and, very rarely, the appearance of novel structures [14]. Very few gangliosides are truly tumor-specific, as many of these altered gangliosides are also found in healthy cells, albeit at a different expression level [25]. Ganglioside expression in tumor cells even seems to vary according to the tumor stage, as metastatic tumors tend to have a different ganglioside profile compared to primary tumors [26, 27]. Besides that, gangliosides are also shed into the TME in much larger quantities than under healthy conditions (an overview is given in Table 1). Gangliosides can even be detected in the blood circulation of cancer patients, as first detected in the plasma of neuroblastoma patients [28, 29], potentially providing a clinically useful biomarker for diagnosis and/or prognosis of tumor recurrence and progression [30].

**Table 1:** Overview of gangliosides expressed and shed by different tumor types

Cancer type	Gangliosides	Refs
Tumors of the central nervous system (glioblastoma, neuroblastoma, medulloblastoma, retinoblastoma)	GM3, GM2, GM1, GD3, GD2, GD1a	[9, 25, 30–40]
Melanoma	GM3, GD3, GD2, GM1	[9, 41–44]
Breast cancer	GM3, GD3, GD2, GD1a, GD1b, GT1b, GQ1b	[9, 45–47]
Lung cancer	GM3, GD3, GM2, GD2, (fucosyl-)GM1	[16, 48–51]
Ovarian cancer	GD3, GD1a	[52, 53]
Renal carcinoma	GM3, GM2	[54, 55]
Colorectal cancer	GM3, GM1, GD1a	[56, 57]
Leukemias	GM3, GD3	[58, 59]
Pancreatic cancer	GM2	[60]
Hepatocellular carcinoma	GM3, GM2, GD3	[61–63]

The expression level of gangliosides in different tissues and tumor types mainly depends on the level of expression of glycosyltransferases involved in their biosynthesis, such as the ST3 β-galactoside α2-3 sialyltransferase 5 (ST3Gal5) which is the enzyme generating monosialylated gangliosides (Fig. 1). ST3Gal5, also named GM3 synthase, is differentially expressed in multiple tumor types compared to adjacent healthy tissue, and associated with beneficial or poor prognosis, depending on the type of tumor. [64–68]. Also, ST8Sia I, which synthesizes disialylated ganglioside species, plays a role in proliferation, invasion and survival of tumor cells *in vitro* [69–71]. ST8Sia1 is highly expressed in melanoma and breast cancer, and is correlated with increased biosynthesis of the downstream gangliosides and poor clinical outcome [72–74]. Inhibition of GD3 synthase (ST8Sia1) *in vivo* suppressed tumor growth and angiogenesis by downregulating vascular endothelial growth factor (VEGF) [75]. For additional information on the synthesis and degradation of tumor-associated gangliosides in tumors, we refer the reader to Groux-Degroote *et* *al*. [16].

The role that gangliosides play in the tumor context is quite diverse as several gangliosides promote tumor cell growth, whereas others stimulate angiogenesis and metastasis [66, 76]. These effects may be for a large part due to the ability of gangliosides to modulate RTK signaling [77]. RTKs often bind growth factors, which upon engagement of the receptor stimulate cell survival, proliferation, differentiation, and migration. Gangliosides can directly engage RTKs within glycolipid-enriched microdomains in the plasma membrane [78]. An aberrant ganglioside profile will thus alter the structure of the glycolipid-containing microdomains and the interaction with RTKs, often resulting in receptor dysregulation, favoring tumor progression. In addition, the shed gangliosides have been shown to influence anti-tumor immunity [79]. In the next sections, we will highlight the immune modulatory effects of gangliosides in the TME.

## Immunomodulatory properties of gangliosides in the tumor microenvironment

The shedding of gangliosides by tumor cells is mainly suppressive to allow tumor cells to escape immune recognition, although the opposite effect has been observed as well, depending on the type and concentration of individual gangliosides [80]. To date, our knowledge regarding immune modulatory properties of the gangliosides shed by cancer cells is still mainly based on *in vitro* studies. The impact of gangliosides on individual immune cell subsets will be further specified below. Of note, in most studies, “soluble” gangliosides are added to the cells; therefore, the observed effects are mainly representative of gangliosides shed by tumor cells, likely forming micelles or vesicles, and does not necessarily reflect how tumor-associated gangliosides modulate anti-tumor immunity.

### T cells

**Fig. 2:**
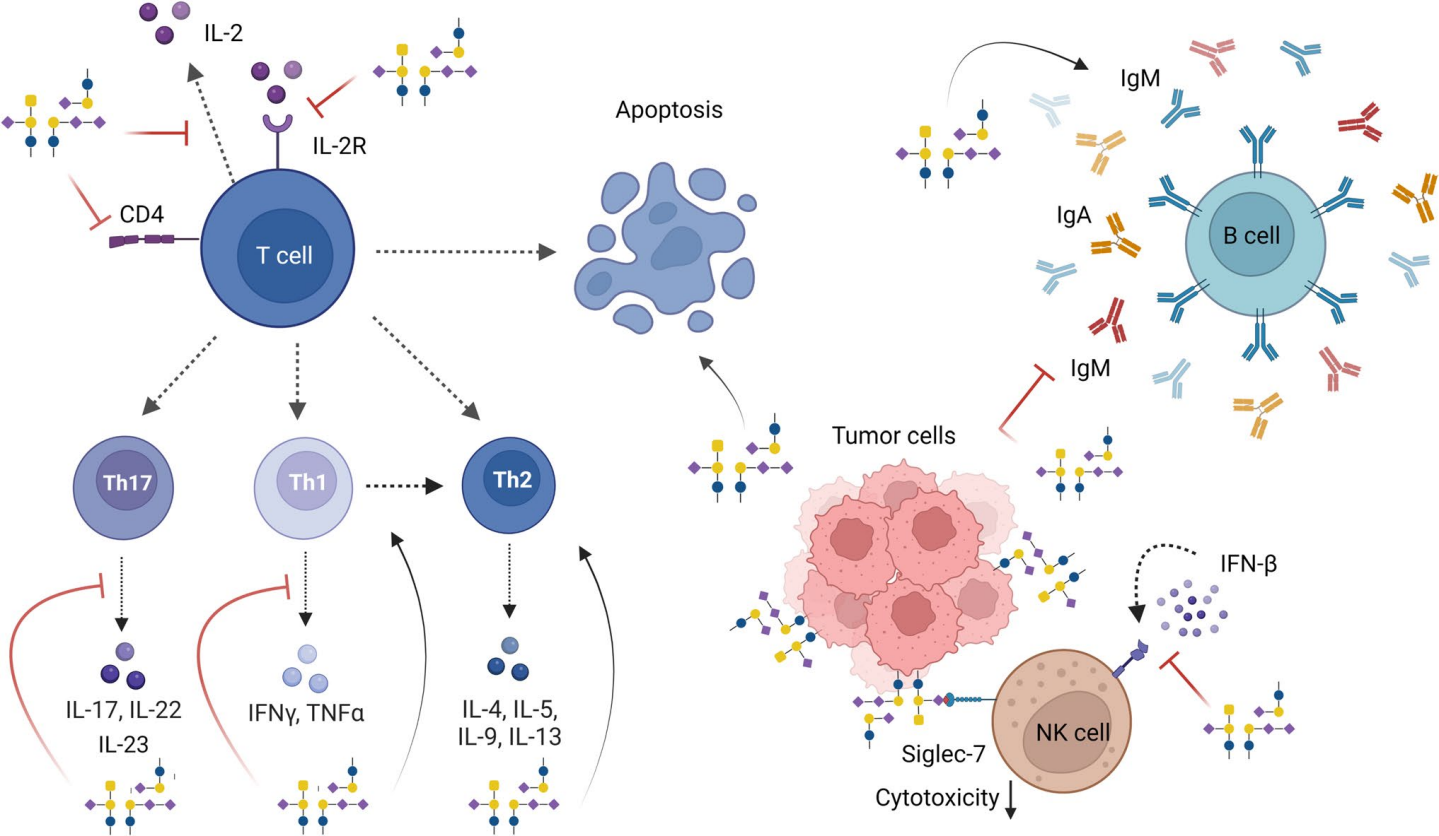
Immunomodulatory effects of gangliosides on lymphoid cells. Gangliosides modify T cell function by different pathways, promoting T cell apoptosis and skewing to a Th2 phenotype. They can also suppress the cytotoxicity of NK cells via Siglec-7 binding and by blocking the interaction between IFN-β and its receptor. In the case of B cells, gangliosides can either stimulate or inhibit the secretion of IgA, IgM, and IgG, depending on the type of ganglioside. General ganglioside structures are depicted in the figure. Please refer to the main text and tables for the action of individual gangliosides. Created with Biorender.com

CD8^+^ cytotoxic T lymphocytes (CTLs) are crucial cells in the anti-tumor immune response, because of their direct cytotoxic action on cancer cells. However, CD4 T helper cells are gaining attention for their direct anti-tumor action as well as their ability to license and sustain CTLs for killing [81]. How gangliosides affect T cell immunity is described below and summarized in Table 2 and Fig. 2.

**Table 2:** Ganglioside effects on the immune system

Ganglioside	Tumorigenic effect	Impact on immune cell/response	References
T cells			
GM3	Pro-tumor	Inhibition of T cell proliferation by blocking the interaction of IL-2 with IL-2R Generation of a Th2 bias through induction of IL-10, limiting a Th1 response Down-regulation of CD4 expression on T cells	[82–84]
GM2	Pro-tumor	Stimulation of T cell proliferation by enhancing the IL-2 response Generation of a Th2 bias by inhibiting Th1 cytokine production and by inducing IL-4 secretion Induction of T cell apoptosis	[54, 83, 85, 86]
GM1	Pro-tumor	Inhibition of T cell proliferation by blocking the interaction of IL-2 with IL-2R Generation of a Th2 bias through induction of IL-10, limiting a Th1 response Induction of T cell apoptosis by inhibiting NF-kB Down-regulation of CD4 expression on T cells	[83, 86–88]
GD3	Pro-tumor	Inhibition of T cell proliferation by neutralizing IL-2 Inhibition of T cell activation through TCR arrest Generation of a Th2 bias by inhibiting Th1 cytokine production Suppression of Th17 activity and IL-17A production Induction of (activated) T cell apoptosis by inhibiting NF-kB, stimulating ROS production, and through induction of caspase-3 and -9	[82, 86, 89–92]
GD2	Anti-/pro- tumor (tumor type dependent)	Stimulation of T cell proliferation by enhancing the IL-2 response Generation of a Th2 bias by inhibiting Th1 cytokine production	[82, 86]
GD1a	Pro-tumor	Inhibition of T cell proliferation by depleting IL-2 Generation of a Th2 bias by inhibiting Th1 cytokines, through induction of IL-10, and by increasing apoptosis of Th1 cytokine-producing cells Induction of T cell apoptosis by inhibiting NF-kB	[83–85, 87, 93]
GD1b	Anti-/pro-tumor (tumor type dependent)	Inhibition of T cell proliferation by depleting IL-2 Generation of a Th1 response by stimulating Th1 cytokine secretion and reducing Th2 cytokines	[83, 93–96]
GT1b	Anti-/pro-tumor (tumor type dependent)	Inhibition of T cell proliferation by binding and blocking IL-2, and interfering with IL-4 Generation of a Th1 response by stimulating Th1 cytokine secretion and reducing Th2 cytokines	[94–97]
B cells			
GM2	Pro-tumor	Inhibition of Ig production by inhibiting IL-10 and TNF-α production	[98]
GD1a	Anti-tumor	Enhancement of Ig production through increased IL-6 and IL-10 production	[99]
GD1b	Pro-tumor	Inhibition of Ig production through reduced IL-6 and IL-10 production	[100]
GT1b	Pro-tumor	Inhibition of Ig production through reduced IL-6 and IL-10 production	[101]
NK/NKT cells			
GM3	Pro-tumor/ anti-tumor	Inhibition of NK cell cytotoxicity (in serum) Cell-associated gangliosides may activate NK cells	[102, 103]
GM2	Pro-tumor	Inhibition of NK cell cytotoxicity	[102]
GM1	Pro-tumor	Inhibition of interferon responsiveness	[104]
GD3	Pro-tumor/ anti-tumor	Reduction of NK cell cytotoxicity (in serum) in a Siglec-7 dependent manner Cell-associated gangliosides may activate NK cells Induction of NK cell immunosuppression Prevention of NKT cell activation	[52, 103, 105–108]
Dendritic cells			
GM3	Pro-tumor	Diminished expression of costimulatory molecules Reduced production of pro-inflammatory cytokines upon LPS stimulation Impaired ability to stimulate allogenic T cell responses Blunted maturation and migration of LC Increased DC and LC apoptosis	[41, 109–111]
GM2	Pro-tumor	Impaired moDC differentiation from monocytes Reduced endocytic capacity Impaired ability to stimulate allogenic T cell responses	[112]
GM1	Pro-tumor	Inhibition of TLR signaling Reduced production of pro-inflammatory cytokines Impaired ability to induce (murine) Th1 responses	[93, 113]
GD3	Pro-tumor	Diminished expression of costimulatory molecules Reduced production of pro-inflammatory cytokines upon LPS stimulation Impaired ability to stimulate allogenic T cell responses Blunted maturation and migration of LC Increased DC and LC apoptosis	[41, 109, 111]
GD2	Pro-tumor	Compromised DC differentiation form murine bone marrow precursor or human CD34^+^ cells Impaired ability to stimulate allogenic T cell responses	[114]
GD1a	Pro-tumor	Inhibition of TLR signaling Diminished expression of costimulatory molecules Reduced production of pro-inflammatory cytokines upon LPS stimulation Impaired ability to induce (murine) Th1 responses Impaired ability to stimulate allogenic and TT-specific T cell responses	[93, 113, 115, 116]
Macrophages			
GM3	Pro-tumor	Inhibition of Fc receptor expression to reduce phagocytosis of tumor cells Suppression of RNI and NO production Inhibition of IL-1β, IL-6, and TNF-α production stimulating tumor growth	[117–120]
GM2	Pro-tumor	Inhibition of Fc receptor expression to reduce phagocytosis of tumor cells Inhibition of TNF-α production stimulating tumor growth	[117, 120]
GM1	Pro-tumor	Inhibition of IL-1β production to counteract cytotoxicity to tumor cells Inhibition of TNF-α production stimulating tumor growth Stimulation of an M2 macrophage bias to support angiogenesis and anti-inflammatory conditions	[117, 120, 121]
GD3	Pro-tumor	Inhibition of IL-1β production to counteract cytotoxicity to tumor cells Inhibition of TNF-α production stimulating tumor growth	[117, 120]
GD1a	Pro-tumor	Suppression of RNI and NO production Suppression of pro-inflammatory cytokine production, including TNFα, IL-1α and IL-1β	[120, 122]
Monocytes			
GM3	Pro-tumor	Inhibition of Fc receptor expression	[117, 123]
GM2	Pro-tumor	Inhibition of Fc receptor expression	[117]
GM1	Pro-tumor	Decreased TLR signaling Suppression of IL-1 production	[93, 117]
GD3	Pro-tumor	Suppression of IL-1 production	[117]
GD1a	Pro-tumor	Downregulation of CD80 and CD40 Impaired production of IL-12 and TNFα Decreased TLR signaling	[93, 124]

*Abbreviations*: *Th1* T helper cell 1, *Th2* T helper cell 2, *Th17* T helper cell 17, *DC* dendritic cell, *LC* Langerhans cell, *IL* interleukin, *TNF* tumor-necrosis factor, *NF-kB* Nuclear Factor kappa B, *ROS* reactive oxygen species, *NO* nitric oxide, *RNI* reactive nitrogen intermediates, *Ig* immunoglobulin, *IFNγ* interferon γ, *TLR* toll-like receptor, *LPS* lipopolysaccharide, *TT* tetanus toxoid

#### Inhibition of T cell proliferation

Gangliosides can inhibit T cell function in multiple ways to promote tumor progression [79]. Most studies demonstrate that gangliosides can interfere with lymphocyte proliferation by depleting IL-2 in a dose-dependent manner, preventing it from binding to the IL-2 receptor (IL-2R) on the surface of activated T cells [82, 83, 125, 126]. However, there is also evidence that gangliosides can bind to IL-2 directly, thus neutralizing free IL-2 without blocking the interaction between IL-2 and the IL-2R [127]. Nevertheless, the presence and position of the sialic acids on the gangliosides seem to be relevant, as GM3 and GD3 have an inhibitory effect on IL-2, while GM2 and GD2 stimulate T cell proliferation by enhancing the response to IL-2 [82]. Moreover, the ability of gangliosides to induce immunosuppression via the IL-2/IL-2R axis appears to be most effective in low protein conditions, since they are known to interact with various serum components like albumin. Therefore, gangliosides will be more immunosuppressive in the local TME where the overall protein concentration is lower than in an *in vitro* setting [126]. In addition, GD1b, and to a lesser extent GM2 and GD3, also inhibit T cell proliferation by interfering with IL-4 signaling even though this effect is much weaker compared to IL-2 [94].

#### Induction of T cell apoptosis

Several studies have demonstrated that depriving proliferating T cells from IL-2 results in T cell apoptosis [128, 129]. In consequence, if gangliosides are able to deplete IL-2, they might indirectly induce T cell death. Moreover, IL-4 is able to trigger apoptosis in an IL-2 dependent manner; thus, a Th2 bias could also result in increased T cell apoptosis [130]. Yet gangliosides are also capable of inducing T cell apoptosis directly.

Even at relatively low concentrations, GM2 is able to induce apoptosis in human T cells *in vitro*. This T cell death was partially blocked by anti-GM2 antibodies, suggesting that GM2 may not be the only ganglioside or the factor involved [54]. Indeed, glioblastoma cell lines expressing both CD70 as well as elevated levels of both GM2 and GD1a are especially prone promotors of T cell apoptosis [85]. The renal cell carcinoma-derived gangliosides and GD3 can enhance apoptosis of human activated T cells *in vitro* through inhibition of the NF-kB pathway, resulting in reduced expression of anti-apoptotic genes, but also through an increase in mitochondrial permeability, accumulation of ROS and cytochrome *c* release [55, 87, 89, 90]. GD3-containing exosomes, isolated from human ovarian tumor ascites, mediated arrest of T cell activation after a short exposure to the ganglioside, but did not result in T cell apoptosis, suggesting that a prolonged exposure to gangliosides is needed to induce T cell death. Also, this GD3-mediated arrest appeared to be dependent on the sialic acid groups, as their enzymatic removal reversed the inhibitory effect [91].

#### Downregulation of CD4 expression

Another mechanism through which gangliosides modulate T cell functionality is by downregulating the surface expression of CD4. Gangliosides GM3 and GM1 inhibit the expression of CD4 in murine and human T lymphocytes, especially in naive T cells, by changing the molecular orientation of CD4 within the cell membrane, which renders CD4 epitopes inaccessible [88, 131, 132]. After this redistribution, CD4 molecules are endocytosed and degraded, resulting in a persistent low level of CD4 expression on the cell surface [133]. Nevertheless, the internalization of CD4 seems to be reversible, since its expression is restored after removal of gangliosides [131]. CD4 modulation appears to be a general characteristic of gangliosides as both the lipid and the sialylated moieties are key in regulating this change in CD4 redistribution [88]. Nevertheless, downregulation of CD4 in the TME could be a long-lasting effect induced by tumor cells to promote tumor progression.

#### Switch from Th1 to Th2

While generally Th1-type responses are favorable in anti-tumor immunity, gangliosides shed by tumors strongly induce Th2 skewing through several mechanisms. They can downregulate Th1 responses by initiating apoptosis of IFNγ-producing cells and thereby inducing a type 2 bias [128]. Furthermore, the presence of gangliosides during T cell activation blocks IL-2 and IFNγ gene transcription without inhibiting the production of Th2-associated cytokines in a human and murine setting [86, 134]. This is possible due to the ability of gangliosides to interfere with NF-κB activation, a transcription factor for pro-inflammatory cytokines, such as IFNγ and IL-2 [134]. Interestingly, the ganglioside-mediated enhancement of IL-4 production *in vivo* was independent of IFNγ, suggesting multiple mechanisms disturbing Th1/Th2 skewing [86]. However, Rayman *et al*. report that GD1a inhibits IFNγ secretion without affecting secretion of type 2 cytokines after human T cell stimulation *in vitro* [128]. Besides, gangliosides GD1a and GM3 strongly induce IL-10 secretion in human T cells, which in turn suppress the production of Th1 cytokines [84].

Even though the majority of gangliosides appears responsible for creating a Th2 bias, some gangliosides seem to induce the opposite. Kanda *et al*. demonstrated that GD1b, and other gangliosides containing even more sialic acids, enhanced the production of IL-2 and IFNγ, while reducing the production of IL-4 and IL-5 in human T cells [95]. This suggests that the ganglioside composition, and specially the sialic acid content, determines whether T cell skewing is towards a Th1 or Th2 type immune response.

#### Th17 interference

The role of Th17 cells in cancer still remains controversial since they exhibit both pro- and anti-tumorigenic activities. Not much is known about how gangliosides modulate Th17 responses in cancer, but GD3 has been shown to suppress the Th17 activity of benign T cells in cutaneous T cell lymphoma [92].

### B cells

Even though T cells are considered to be the most effective immune cells mediating the anti-tumor immune response, there is increasing evidence that B cells play an important role in tumor control. Besides producing antibodies, tumor-infiltrating B cells can present antigens to T cells and secrete a variety of cytokines [135]. However, tumor-infiltrating B cells are also able to promote angiogenesis or secrete immunoregulatory cytokines, including TGFβ and IL-10, that suppress the anti-tumor immune response and its effector cells [136].

#### Immunoglobulin production

Gangliosides have been shown to modulate immunoglobulin (Ig) secretion of B cells (Table 2; Fig. 2). For example, GM2 can inhibit the production of IgM, IgA and IgG in human B cell lines. This effect was counteracted by the addition of both IL-10 and TNFα, suggesting that GM2 inhibits Ig production through the inhibition of IL-10 and TNF-α secretion [98]. Similarly, GD1b and GT1b also suppress IgM, IgG, and IgA production from human PBMCs by reducing IL-6 and IL-10 release of CD4^+^ T cells and monocytes, respectively [100, 101]. In contrast, the ganglioside GD1a enhances IgG, IgM, and IgA production of human PBMCs by increasing IL-6 and IL-10 secretion of monocytes [99]. In all these *in vitro* studies, the effect of gangliosides on Ig production was reversible and did not affect the proliferation nor the viability of the B cells [98–101]. So, gangliosides may be involved in regulating humoral responses, but how they modulate B cells in the TME remains unclear.

### NK and NKT cells

Natural killer (NK) cells are important players in the overall immune responses against tumors. Their functions are similar to that of cytotoxic T cells, considering they induce anti-viral and anti-tumor immunity by producing IFNγ, granzyme B, and perforin, resulting in cell lysis. They are able to recognize and eliminate MHC-I deficient cells (missing-self) or through engagement of tumor-specific antibodies with their CD16 Fc receptor, triggering antibody-dependent cell-mediated cytotoxicity (ADCC) [137]. Gangliosides can actively suppress NK cell function, decreasing their cytotoxic response and cytokine production (Table 2; Fig. 2).

#### Inhibition of NK cell cytotoxicity

GM3 and GM2 gangliosides isolated from human brain tissue were able to inhibit NK cell activity *in vitro*, in contrast to other gangliosides containing more sialic acids. GM3 and GM2 are present in high concentrations in neuroblastoma and gliomas, supporting the hypothesis that shedding of these gangliosides promotes tumor progression [102]. Also, tumor cells containing GD3 decreased the cytotoxic ability of NK cells in an *in vitro* model [105]. Interestingly, incubation of lymphoma cells with gangliosides prior to NK cell co-culture, resulted in increased NK cell activity towards tumor cells. This suggests that tumor-bound gangliosides function as target structures recognized by NK cells, while shed gangliosides actually contribute to NK cell inhibition during tumor development [103]. One possible explanation might be the insertion of gangliosides into the plasma lipid bilayer of tumor cells, leading to new binding sites for NK cells, thereby increasing their capacity to kill tumor cells.

One of the mechanisms used by gangliosides to modulate NK cell cytotoxicity could be via the binding of sialic-acid binding immunoglobulin-like lectins (Siglecs). Siglecs are a family of sialic acids receptors expressed on immune cells, most of them containing an immunoreceptor tyrosine-based inhibitory motif (ITIM). Nicoll *et al*. demonstrated that GD3-expressing cells strongly bind to Siglec-7 on NK cells, thereby downregulating NK cell cytotoxicity [106] (Fig. 2). In another study, blocking Siglec-7 binding with an anti-GD2 antibody also sensitized tumor cells to macrophage-mediated phagocytosis resulting in tumor eradication *in vivo* [138].

#### Inhibition of interferon production

Several interferons, including IFN-β, have been shown to enhance the cytotoxic activity of NK cells, and are therefore important in anti-tumor immune responses. The gangliosides GM1, GD1b, and GT1b all inhibit the stimulatory effect of IFN-β on murine NK cells *in vitro*, by competing with NK cells for their interaction with IFN-β. Likely, the direct binding of gangliosides is responsible for the IFN-β-mediated suppression of NK cell activation [104]. The suppression of IFNs is thus a direct mechanism of action of gangliosides to stimulate tumor progression and enhance immune evasion.

#### Immunosuppression due to senescence

A recent study revealed that senescent cells modify their glycosphingolipid composition towards a higher ganglioside level, characterized by the overexpression of GD3. This is due to a transcriptional upregulation during senescence of the gene encoding the enzyme ST8Sia1, which is responsible for GD3 synthesis. Increased levels of GD3 lead to an immunosuppressive effect on NK cells *in vitro* and *in vivo* by binding to Siglec-7 receptor on NK cells [107].

#### NKT cells

The effect of gangliosides on NKT cells is poorly studied. However, Wu *et al*. demonstrated that mice immunized with GD3^+^ human melanoma cells developed a CD1d-restricted NKT cell response against GD3. The observed response was typical of Th2-like cells (secretion of IL-4, IL-10), although the cells also produced some transient IFNγ [108]. Similarly, GD3 isolated from ovarian cancer-associated ascites also prevented activation and IFNγ production by NKT cells; however, in this study, also, IL-4 secretion was inhibited [52].

### Dendritic cells

**Fig. 3:**
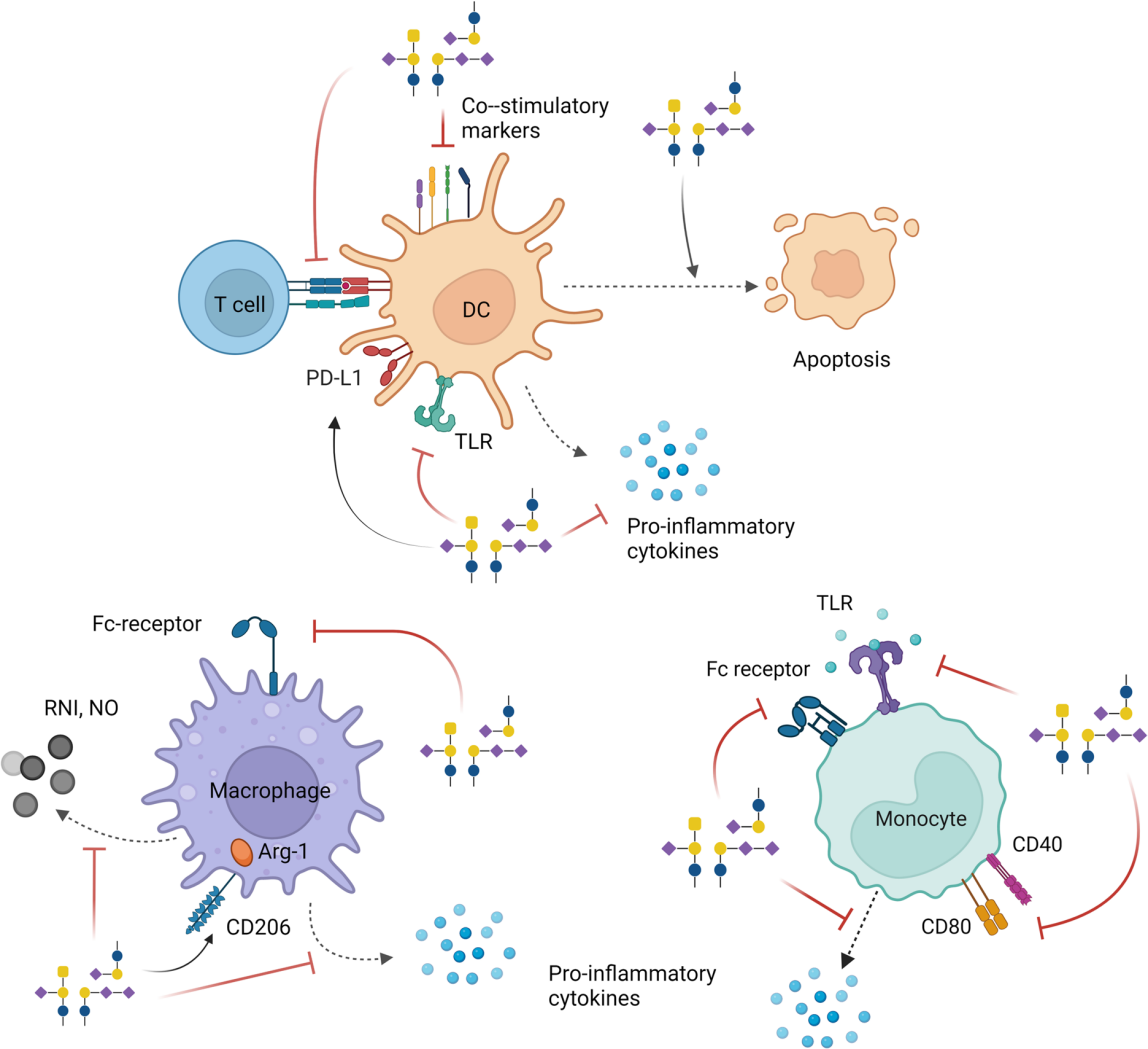
Immunomodulatory effects of gangliosides on myeloid cells. Gangliosides downregulate the differentiation and maturation of DCs through several mechanisms. They reduce the capacity of DCs to activate T cells and induce DC apoptosis. Gangliosides inhibit the production of pro-inflammatory cytokines by DCs, macrophages, and monocytes. Besides, they can block Fc receptors, TLRs, and co-stimulatory molecules on different myeloid populations. Some gangliosides stimulate the M2-polarization of macrophages by increasing the levels of Arg-1 through CD206, and suppress the production of RNI and NO. General ganglioside structures are depicted in the figure. Please refer to the main text and tables for the action of individual gangliosides. Created with Biorender.com

Dendritic cells (DCs) are professional antigen-presenting cells, capable of initiating adaptive T cell responses towards pathogens and malignant cells. In addition, DCs play a crucial role in maintaining immunological tolerance, through the selection and depletion of self-reactive T cells. Gangliosides seem to disrupt the whole DC life cycle, ranging from DC development to DC maturation, thus promoting a tolerogenic TME (Table 2; Fig. 3).

#### Impaired DC differentiation

*In vivo* DCs develop from dedicated precursors; however, under inflammatory conditions, monocytes can also differentiate into so-called monocyte-derived DCs (moDCs). The impact of ganglioside on DC differentiation has not been widely studied; however, early reports indicate that neuroblastoma-derived GD2 impairs DC development from mouse bone marrow and human CD34^+^ progenitors [114], while GM2, GD3, and GM3 dampen monocyte to DC differentiation, resulting in a DC population with altered morphology, reduced endocytic capacity, as well as a reduced expression of the DC markers CD1a, HLA-DR, and CD80 [41, 112].

#### Inhibition of TLR activation

Interestingly, in non-small cell lung cancer, a high expression of the ganglioside N-glycolyl-GM3 was associated with a decreased density of mature CD83^+^ DCs, indicating that gangliosides may blunt DC maturation [48]. Indeed, in several *in vitro* studies, DC maturation was inhibited in the presence of gangliosides. DC maturation is generally triggered by the engagement and subsequent signaling through pattern recognition receptors, such as the toll-like receptors (TLRs). The gangliosides GM1, GD1a, and GD1b are able to inhibit the activation of multiple TLRs *in vitro* through the upregulation of the TLR signaling pathway inhibitor, IL-1 receptor–associated kinase M (IRAK-M) [93]. GD1a also reduces TLR-dependent phosphorylation of p38, the key downstream kinase in TLR4 signaling [115], and the subsequent nuclear translocation of NF-κB [116, 124]. This inhibition of TLR activation occurs in a dose-dependent manner and seems to be reversible, with complete recovery of TLR signaling, as assessed by the regained production of pro-inflammatory cytokines, after removal of gangliosides [93]. Interestingly, GD3 is furthermore able to block CD40L-induced maturation [41].

#### Interference with DC maturation

Gangliosides appear to have the ability to inhibit the LPS-driven and IFNγ boosted DC maturation and cytokine production, and therefore downregulate the pro-inflammatory response triggered against tumor cells.

Gangliosides GM3 and GD3 are modest inhibitors of human DC maturation marker expression *in vitro*, influencing costimulatory molecules CD40, CD80, CD86, and MHC-II [109]. While GM3 only impaired IL-10 and IL-12 production, GD3 also reduced IL-6 and TNFα secretion after LPS triggering [109]. A similar GM3-mediated downregulation of CD40 and IL-12 was observed *in vivo* in neuroblastoma bearing mice [110]. In these mice, IL-12 secretion was abrogated in a CD40-dependent manner. GD1a and to a lesser extent GM1 also inhibit the TLR-induced expression of co-stimulatory molecules, such as CD40, CD80, CD83, and CD86 on murine and human DC *in vitro* [113, 116, 124]. Moreover, GD1a is a potent inhibitor of IL-6, IL-10, IL-12, and TNFα secretion *in vitro* [113, 116, 124]. Strong pro-inflammatory conditions might counteract the tumor ganglioside-induced phenotype. Yet, IFNγ further amplified the GD1a effects and augmented both IDO1 and PD-L1 expression on the DCs, indicating that IFNγ and GD1a may act in concert to further amplify the tumor immunosuppressive loop [115].

#### Inability to activate T cells

Exposure of DCs to a wide variety of gangliosides not only impairs TLR-mediated maturation, costimulatory marker expression, and cytokine production, but it also impairs their ability to activate T cells. Treatment of both murine and human DCs with a plethora of gangliosides, including GM3, GD3, GM2, GD2, and GD1a, reduces the DC stimulatory capacity *in vitro*, resulting in a diminished T cell proliferative response in an allogeneic mixed lymphocyte reaction (MLR) assay [41, 109, 112, 114, 116]. GD1a-preincubated DCs show a similar deficiency in stimulating tetanus toxoid antigen-specific CD4^+^ T cell responses [124].

In addition, and expected from the aberrant cytokine secretion profiles, gangliosides can derail DC-mediated induction of T helper differentiation. Both GD1a and GM1 compromise Th1 and Th2 differentiation and instead favor the instruction of functional Tregs [113]. Interestingly, this altered Th1 skewing could not be rescued by exogenous IL-12, suggesting that other factors, besides IL-12, contribute to the tapered Th1 differentiation.

#### Impaired maturation and migration of LC

Langerhans cells (LC) are a type of dendritic cells present in the epidermal layer of the human skin where they act as an immune barrier. Melanoma-derived GM3 and GD3 impair the maturation and migration of human epidermal LCs, which might explain the marked decrease in activated LCs in the lymph nodes close to the tumor [111]. GM3 and GD3 both significantly downregulate expression of costimulatory and maturation markers, which correlated to an impaired ability of the LCs to mount allogeneic T cell proliferation. Also, expression of CCR7 was downregulated, reducing the migration towards CCL19, a chemokine crucial for LC migration to the lymph node.

#### Induction of DC and LC apoptosis

The gangliosides GM3 and GD3 are also able to induce early apoptosis of DCs and LCs (Fig. 3) [111, 139], which may be attributed to a dysregulated and early DC differentiation and maturation [41]. Interestingly, the GM3- and GD3-induced apoptosis was independent of ganglioside catabolism [139], but did involve activation of caspase-3. Whereas GD3 exposure let to a loss of mitochondrial membrane potential and production of ROS, GM3 exposure did not [139]. Clearly, the mechanisms through which GM3 and GD3 induce caspase-3 activation and apoptosis are different and still not fully elucidated.

Overall, gangliosides seem to foster the development of an immunosuppressive tumor microenvironment, low in fully matured DCs and elevated in Treg numbers.

### Macrophages

Macrophages are important players of the innate immune system. They are not a single-cell population with a defined phenotype and function, but depending on the tissue context rather a collection of cell types with a wide range of functional roles in homeostatic and pathological conditions [140]. In the context of cancer, myeloid cells are often seen as double-edged swords, since on the one hand, they have the potential to kill tumor cells, mediate ADCC, and activate lymphoid cells. In contrast, tumor-associated macrophages (TAMs) contribute to cancer progression and angiogenesis as well as to an immunosuppressive TME [141]. The effects of specific gangliosides on macrophages can be found in Table 2 and Fig. 3.

#### Inhibition of Fc receptor expression

The Fc receptor on macrophages is an important contributor to the immune functions of macrophages [140, 142]. It can bind specifically to antibody-opsonized malignant cells, thereby resulting in phagocytosis of tumor cells [143]. Treatment of human macrophages with GM3 and GM2 gangliosides *in vitro* inhibited their Fc receptor expression, while other gangliosides did not have this capacity [117].

#### Suppression of cytokine release

IL-1β is produced by infiltrating myeloid cells, including macrophages, and can promote tumor progression, metastasis, and the generation of an immunosuppressive TME [144]. GM1 and GD3 melanoma-derived gangliosides inhibit IL-1β production by macrophages *in vitro* [117]. Similarly, GM3 suppresses the secretion of IL-1β and IL-6 in murine RAW 264.7 macrophages [145]. The production of IL-1α and IL-1β was also inhibited by GD1a in LPS-stimulated macrophages [122]. Even though gangliosides might thus be involved in dampening IL-1β release, the underlying mechanisms are still unclear.

TNFα is a pro-inflammatory cytokine that mainly has anti-tumorigenic effects; however, at low levels, this cytokine may also sustain tumor development [146]. Several gangliosides, including GM3 and GD1a, were all effective in reducing TNFα production by murine macrophages *in vitro* [118, 122]. These gangliosides appear to act at an early step of the signaling transduction cascade as they inhibit MAPK upstream of NF-kB, a key transcription factor for TNFα [118].

#### Inhibition of RNI production

Reactive nitrogen intermediates (RNI), including nitric oxide (NO), are produced by TAMs and can cause DNA damage and genomic instability, resulting in tumor progression [141]. In contrast, RNI produced by macrophages may also be involved in tumor killing [147]. GM3 and GD1a, amongst other gangliosides shed by tumor cells, appear to inhibit the production of RNI and NO by macrophages *in vitro* [119, 122, 145]. The mechanism behind this inhibition is still unresolved, but likely involves a direct action of the gangliosides on macrophage function.

#### Stimulation of M2 macrophage polarization

Macrophages can adopt different functional phenotypes, classically referred to as the pro-inflammatory M1-like macrophages and the anti-inflammatory M2-macrophages, which share similarities with TAMs in the TME. GM1 seems to be a potent stimulator of the polarization towards M2-like macrophages *in vitro* for both human as well as murine macrophages. It does so by increasing the expression of arginase-1 (Arg-1), a M2-macrophage marker, through the mannose receptor (CD206) and common gamma chain (γc)-mediated activation of JAK3 and STAT6 [121]. In addition, GM1-stimulated macrophages secrete monocyte chemoattractant protein-1 (MCP-1/CCL2) promoting tumor growth and angiogenesis [121].

### Monocytes

Monocytes are well known as a precursors of macrophages and moDCs, but they can also act as APCs that are able to prime CD8^+^ and CD4^+^ T cells [148]. Similar to other immune cell subsets, gangliosides modulate the proliferation and function of monocytes [149]. For instance, exposing LPS-stimulated monocytes to GD1a downregulates CD40 and CD80, as well as hampers the release of IL-12 and TNFα [120, 124]. Moreover, incubation of monocytes with specific gangliosides impairs Fc receptor expression (GM3 and GM2), reduces IL-1 production (GM1 and GD3), and decreases TLR signaling [93, 117]. Additionally, GM3 suppresses monocyte adhesion to endothelial cells by inhibiting ICAM-1 and VCAM-1 expression on endothelial cells through activation of NF-κB [123] (Table 2; Fig. 3).

### Myeloid-derived suppressor cells (MDSCs)

Tumor-infiltrating MDSCs are a heterogeneous population of myeloid cells known to have many immunosuppressive properties, including recruitment of Tregs and inhibition of CD8^+^ T cell infiltration. To study the role of gangliosides shed by tumor cells, Wondimu *et al*. used a novel tumor cell created by oncogenic transformation of murine embryonic fibroblasts in which the GM3 and GM2 synthase were knocked-out [150]. This KO rendered the tumor cells completely devoid of gangliosides and resulted in decreased tumor growth *in vivo* and less infiltration of MDSCs in the TME. The exact mechanism on how gangliosides enhance MDSC remains unclear; however, it might be due to altered chemokine release. In addition, gangliosides can induce the production of iNOS and Arg-1, which are crucial factors for the MDSC-induced immunosuppression [150].

## Discussion and outlook

Gangliosides play crucial roles in the context of tumor immunity. During malignancy, changes occur in the glycosphingolipid composition present on the surface of tumor cells, which aid in tumor progression through inhibition of the immune system [11]. Cancer cells have the ability to shed gangliosides into the surrounding environment or to present them as tumor-associated antigens influencing a wide range of immune cells, including T lymphocytes, B cells, NK cells, DCs, macrophages, and monocytes. Gangliosides can suppress the activation, proliferation, and cytotoxicity of these immune cells to inhibit the anti-tumor immune response. However, certain gangliosides also have the capacity to activate specific immune cells. This ganglioside-mediated immunomodulatory effect depends on the type of ganglioside, the amount of sialylation, and on the specific tumor type.

Many aspects of ganglioside-mediated immunomodulation are still unclear, especially regarding individual gangliosides and their specific roles during the anti-tumor immune response. Multiple studies have focused on T cells, dendritic cells, and macrophages and demonstrated that most gangliosides have potent inhibitory effects on the activation, proliferation and functionality of these immune cells. Overall, the shedding of gangliosides by cancer cells and their suppressive actions appear to be great weapons to counteract anti-tumor immunity. Interestingly, the impact of these glycosphingolipids on B cells, NK cells, monocytes, and even neutrophils has hardly been investigated. Moreover, most of the studies related to the immunomodulatory properties of gangliosides have been performed *in vitro* with isolated gangliosides in co-cultures with one specific immune cell subset. More elaborate functional assays and *in vivo* studies are needed to gain more insight in the shedding of gangliosides and their immunomodulation in a dynamic and complex environment like the TME.

The gangliosides described in this review can be categorized into gangliosides that exhibit pro-tumorigenic and anti-inflammatory properties stimulating tumor growth, and gangliosides that exhibit anti-tumorigenic and pro-inflammatory properties. Almost all gangliosides, including GM3, GM2 and GM1, are considered immunosuppressive, whereas GD2, GD1b, and GT1b show a dual response depending on the tumor type and the interacting immune cell. Remarkably, gangliosides containing one or two sialic acid moieties appear to be good immune cell inhibitors, thereby promoting tumor progression. On the contrary, the more complex gangliosides, carrying more than two sialic acid residues, also possess anti-tumorigenic features, stimulating T cell proliferation and production of pro-inflammatory cytokines. The mechanism behind this phenomenon is unknown, but could be related to the fact that simple gangliosides predominate in most peripheral tissues [5]. Clearly, the molecular composition of gangliosides is of great importance in cancer immunosurveillance. It is also relevant to mention that sialylated glycosphingolipids are ligands for inhibitory Siglec receptors on immune cells (reviewed in [151]). Siglec-1, for example, interacts with several gangliosides, including GM3, GD1a, GD1b, and GT1b resulting in phagocytosis, degradation, and antigen presentation. Siglec-7 and Siglec-9, amongst other Siglecs, also engage endogenous gangliosides to inhibit immune signaling, such as NK cell cytotoxicity. The ganglioside-Siglec axis could be one of the mechanisms by which lipid sialoglycans modulate immune responses. However, more research is needed to draw strong conclusions about the exact role of ganglioside-Siglec interactions in the TME.

Since many tumors upregulate and shed gangliosides, they are prime candidates for antibody therapy. Gangliosides present in the bloodstream are a perfect diagnostic marker for several tumors and provide targets for antibody therapies directed against tumor-associated gangliosides [16, 152]. Many of these antibodies are now being investigated in pre-clinical and clinical studies [9, 14, 25]. For example, anti-GD2 is currently being administered to neuroblastoma patients in a phase III trial (trial NCT01704716). Additionally, a GD3 antibody-drug conjugate has been administered to melanoma patients in a phase I trial (NCT03159117). However, so far, these therapies were only partially successful. Synthetic antigens mimicking the carbohydrate moiety of GD2 and GD3 gangliosides as vaccines have been tested in an *in vivo* pre-clinical setting. Interestingly, mice vaccinated with these synthetic gangliosides had an initial γδ T cell response followed by a cascade of CD8^+^ T cells that infiltrated tumors [153]. Since these proof-of-concept synthetic gangliosides induce both a cellular and humoral response, these glycomimetic vaccines might be expanded to target other tumor gangliosides expressed in several malignancies [153]. In combination with other treatments or as an adjuvant therapy, this approach could be a promising new lead to combat cancer cells. Nevertheless, exploiting tumor-associated gangliosides has been challenging, because glycolipids are poor immunogens, as the carbohydrates may not be efficiently processed nor presented in the context of MHC-I/II [154]. An alternative could be the development of chimeric antigen receptor (CAR) T- and NKT cells directed against tumor-associated gangliosides. Because of its highly tumor-specific expression pattern, GD2-specific CAR-T- and NKT cells have already been used in neuroblastoma or glioma with promising results [155–157]. The development of GD3 CAR-T cells is in a pre-clinical phase, but showed promising results in several murine tumor models [158].

In conclusion, this review highlights gangliosides and their importance in the complex and dynamic process of tumor development and anti-tumor immunity. The importance of shed- or tumor-associated gangliosides in modulating the anti-tumor immune response is evident and multi-disciplinary in nature. We postulate that future research should focus more on complex sialylated glycosphingolipids and on the Siglec-ganglioside interactions to better understand their physiological role and to expand the horizon for targeting gangliosides as an immunomodulatory strategy to cure cancer.
